# Visualizing cellularity and angiogenesis in newly-diagnosed glioblastoma with diffusion and perfusion MRI and FET-PET imaging

**DOI:** 10.1186/s13550-021-00817-3

**Published:** 2021-08-16

**Authors:** Friederike Liesche-Starnecker, Georg Prokop, Igor Yakushev, Christine Preibisch, Claire Delbridge, Hanno S. Meyer, Kaywan Aftahy, Melanie Barz, Bernhard Meyer, Claus Zimmer, Jürgen Schlegel, Benedikt Wiestler, Jens Gempt

**Affiliations:** 1 Department of Neuropathology, Institute of Pathology, School of Medicine, Technical University Munich, Munich, Germany; 2 Department of Nuclear Medicine, Klinikum rechts der isar, School of Medicine, Technical University Munich, Munich, Germany; 3 Department of Neuroradiology, Klinikum rechts der isar, School of Medicine, Technical University Munich, Munich, Germany; 4 Department of Neurosurgery, Klinikum rechts der isar, School of Medicine, Technical University Munich, Ismaningerstr. 22, 81675 Munich, Germany; 5 TranslaTUM (Zentralinstitut für translationale Krebsforschung der Technischen Universität München), Einsteinstraße 25, Munich, Germany

## Abstract

**Purpose:**

Combining imaging modalities has become an essential tool for assessment of tumor biology in glioblastoma (GBM) patients. Aim of this study is to understand how tumor cellularity and neovascularization are reflected in O-(2-[18F]fluoroethyl)-L-tyrosine positron emission tomography ([18F] FET PET) and magnetic resonance imaging (MRI) parameters, including cerebral blood volume (CBV), fractional anisotropy (FA) and mean diffusivity (MD).

**Methods:**

In this prospective cohort, 162 targeted biopsies of 43 patients with therapy-naïve, isocitrate dehydrogenase (IDH) wildtype GBM were obtained after defining areas of interest based on imaging parameters [18F] FET PET, CBV, FA and MD. Histopathological analysis of cellularity and neovascularization was conducted and results correlated to imaging data.

**Results:**

ANOVA analysis showed a significant increase of CBV in areas with high neovascularization. For diffusion metrics, and in particular FA, a trend for inverse association with neovascularization was found. [18F] FET PET showed a significant positive correlation to cellularity, while CBV also showed a trend towards correlation with cellularity, not reaching significant levels. In contrast, MD and FA were negatively associated with cellularity.

**Conclusion:**

Our study confirms that amino acid PET and MR imaging parameters are indicative of histological tumor properties in glioblastoma and highlights the ability of multimodal imaging to assess tumor biology non-invasively.

## Introduction

Precise tumor characterization by means of modern imaging methods such as magnetic resonance imaging (MRI) and positron emission tomography (PET) is crucial for interdisciplinary decision-making regarding therapy options for glioblastoma (GBM) patients. Therefore, it is of high interest how the tumor’s biology is reflected in the different imaging modalities. Earlier studies have shown that with O-(2-[18F]fluoroethyl)-L-tyrosine([FET) PET, the uptake of amino acid in gliomas and thus, metabolically active tumor cells can be detected [1, 2]. In brain metastases, high [18F] FET PET intensity was indicative of high tumor and low necrosis content [3]. Furthermore, static [18F] FET PET uptake was shown to correlate with neovascularization in GBM [2], which is an essential diagnostic parameter in histopathological analysis [4, 5]. Cerebral blood volume (CBV), an established parameter of perfusion-weighed imaging (PWI), has also been proven to be a promising modality in assessing tumor vascularity [6]. In addition, it has been shown that diffusion tensor imaging (DTI) has better accuracy in detecting tumor progression in GBM compared to conventional MRI [7]. DTI parameter fractional anisotropy (FA), for instance, is decreased in GBM [8], following axonal degeneration [9].

Due to the strong intratumoral heterogeneity—a hallmark of GBM—and sampling bias, it has been difficult to compare MRI findings with the tumor’s histopathology in an exact topographic manner. This prospective cohort accounts for tumor heterogeneity by obtaining targeted biopsies from diverse areas of interest based on comprehensive preoperative imaging. By comparing a number of physiological parameters ([18F] FET PET, CBV, FA and mean diffusivity (MD)) with histopathological analyses of neovascularization and cellularity in newly diagnosed GBM, our study provides important insights into imaging biology of GBM.

## Materials and methods

### Patients

This study was approved by our local ethics committee (284/16S). All 43 patients were part of a prospective glioblastoma cohort from February 2018 to October 2020 and gave written informed consent. The delay between both exams was less than 7 days in all cases, as was the delay between the last imaging and surgery. Only patients with newly-diagnosed, therapy-naïve isocitrate dehydrogenase (IDH) wildtype glioblastoma, WHO CNS grade 4 were included. Neuropathological diagnosis was made according to World Health Organization (WHO) classification, 2016 [5] and reevaluated in the context of this study. Ten patients from this cohort have been reported earlier [13], albeit with a focus of spatial overlap of advanced imaging techniques.

### Image acquisition

[18F] FET PET data were acquired on a PET/MR (Biograph mMR, Siemens Healthcare GmbH, Erlangen, Germany), and a PET/computed tomography (CT) (Biograph mCT; Siemens Healthcare, Knoxville TN, USA), according to a standard clinical protocol. Patients were asked to fast for a minimum of 4 h before scanning. Emission scans were acquired at 30 to 40 min after intravenous injection of a target dose of 185 ± 10% MBq [18F] FET. Attenuation correction was performed according to the vendor’s protocol.

MR imaging was performed on a Philips (Best, The Netherlands) 3 T scanner (Achieva or Ingenia). Our MR protocol included an isotropic FLAIR (voxel size 1 mm^3^, TE = 269 ms, TR = 4800 ms, TI = 1650 ms), isotropic T1-weighted-TFE (voxel size 1mm^3^, TE = 4 ms, TR = 9 ms) before and after contrast, axial T2-weighted TSE (voxel size 0.36 × 0.36 × 4mm^3^, TE = 87 ms, TR = 3396 ms), DTI (spin echo EPI, voxel size 2 × 2 × 2 mm^3^, TE = 78 ms, TR = 5000 ms) with 32 gradient directions (*b* = 800 s/mm^2^) and one non-diffusion-weighted volume, as well as dynamic susceptibility contrast (DSC) perfusion (single shot EPI, voxel size 1.75 × 1.75 × 4 mm^3^, TE = 40 ms, TR = 1547 ms, flip angle = 75°, 80 dynamics). Patients received 0.1 mmol/kg of Gd-containing contrast agent (Gddiethylene-triamine-pentacetate (DTPA) or Dotarem).

### Image processing

Processing of DSC data for relative CBV (rCBV) parameter maps used custom programs [10, 11] in MATLAB R2019b (MathWorks, Natick, MA, USA). Spatial co-registration of the different modalities and segmentation of anatomical images for gray matter (GM), white matter (WM) and cerebrospinal fluid (CSF) were conducted using SPM12 (www.fil.ion.ucl.ac.uk/spm) with standard parameter settings. Leakage‐corrected CBV values were obtained using a reference curve approach and numerical integration. rCBV values were calculated by assuming healthy WM values of 2.5%. DTI data were analyzed using the open-source dipy framework [12]. For estimation of the diffusion tensor, a non-linear least square algorithm was used, and mean MD and FA maps were calculated from the tensor. FET-PET intensities inside the biopsy areas was automatically normalized against the WM background signal as derived above.

### Image analysis

All images and parameter maps ([18F] FET PET, CBV, MD, FA) from a single patient were spatially normalized into the SRI24 atlas space and resampled to 1 mm isotropic resolution using a rigid, mutual information-driven registration with the open-source ANTs software (https://stnava.github.io/ANTs/). Biopsy locations were manually annotated in the images by J.G. and B.W., and median parameter values of each biopsy area in the four parameter maps were automatically extracted using a custom Python script.

### Targeted biopsies

Biopsies were obtained using a cranial navigation system (Brainlab AG, Munich, Germany) and intraoperative neuronavigation. To limit the influence of brain shift, biopsies were obtained before tumor removal at the beginning of surgery with minimal dural opening. Tissue samples were then transferred to 10% buffered formalin and sent to the Department of Neuropathology for further processing and histopathological evaluation.

### Histopathological analysis

After formalin fixation and paraffin embedding, hematoxylin and eosin staining was performed on 2 μm-thick slides. Histopathological analysis was performed by a neuropathologist (F.LS.) not familiar with the results of imaging analysis. Cellularity was determined by counting cell amounts of 1/4 high power field (HPF; ocular tenfold, objective 40-fold) of three randomly chosen regions of each biopsy, as described previously [13]. For statistical analysis, the median value was calculated. Necrotic areas were excluded, endothelial inflammatory cells were not counted. Neovascularization was scored from “no” to “high” (‘no’ meaning no noteworthy vascularization, “low” meaning vessel area < 30% of total biopsy area and “high” meaning vessel area < 30% of total biopsy area).

### Statistical analysis

Spearman's method was used to assess the correlation between the MRI and PET parameter values and cellularity. Analysis of Variance (ANOVA) method was performed to detect differences in parameter values between different levels of neovascularization. Values of *p* < 0.05 were considered statistically significant.

## Results

162 targeted biopsies were obtained from 43 patients (median age 69 y, range 34 to 85 y; 25 male). Per patient, 1 to 6 biopsies were taken (mean 3.8). All parameter maps were available for 78 samples of 23 patients. For 20 patients, [18F] FET PET was missing, whereof for 1 patient, no CBV was available either.

For [18F] FET PET, and CBV, parameter values increased with higher neovascularization. ANOVA revealed a significant increase of CBV in areas with “high” neovascularization (*p* = 0.003). In contrast, there was a trend towards lower FA values in areas with higher neovascularization (*p* = 0.215).

[18F] FET PET was significantly correlated with cellularity (*ρ* = 0.229, *p* = 0.039) and CBV showed a trend towards correlation with cellularity, not reaching significant levels (*ρ* = 0.129, *p* = 0.106). MD and FA were negatively associated with cellularity. For MD, the correlation was borderline significant (*ρ* = −0.154, *p* = 0.050 for MD; *ρ* = −0.095, *p* = 0.231 for FA).

**Fig. 1:**
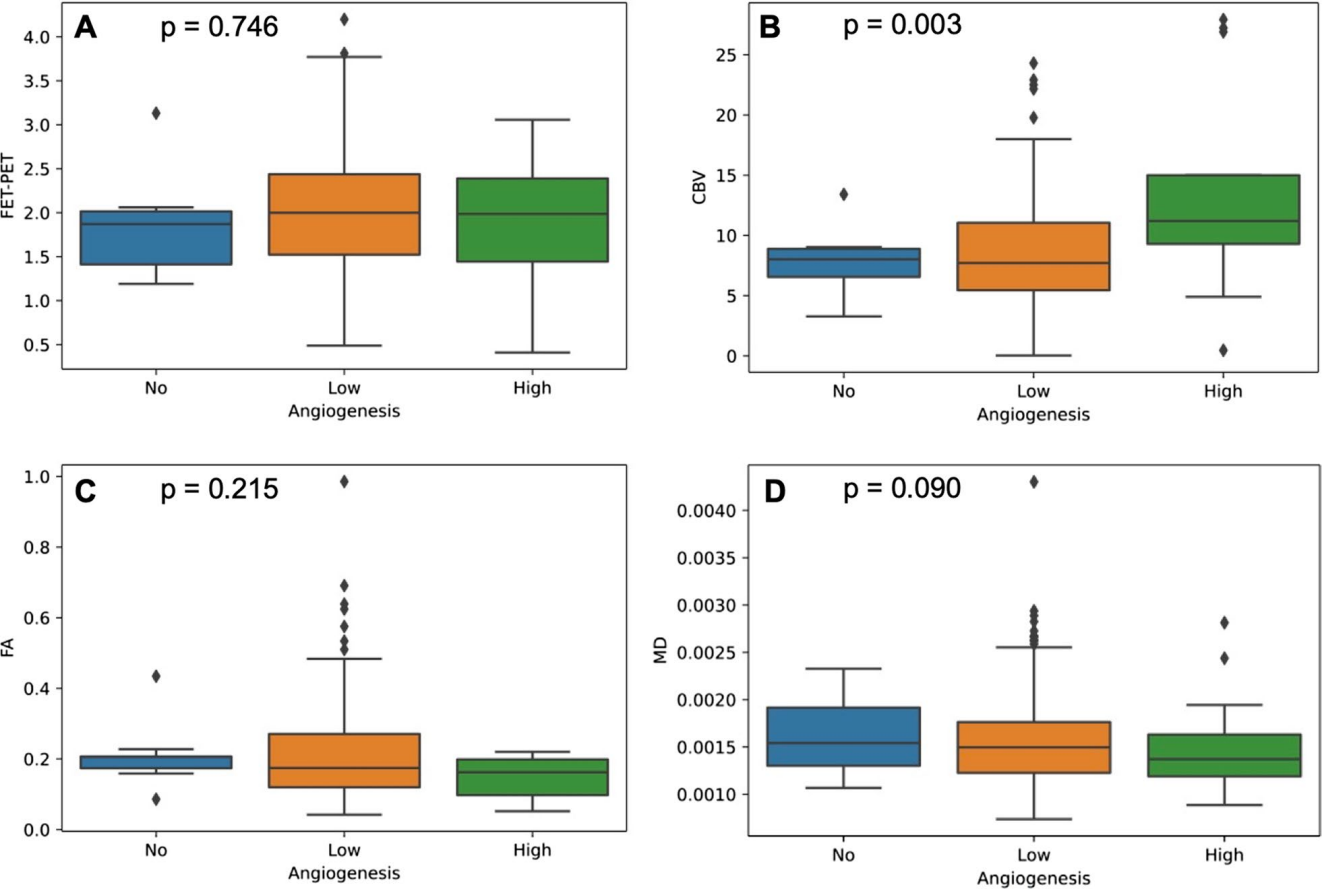
Imaging parameters and neovascularization. Boxplots show median [18F] FET PET (**a**), CBV (**b**), FA (**c**) and MD (**d**) signal in biopsies without (blue), with low (orange), or high (green) neovascularization

**Fig. 2:**
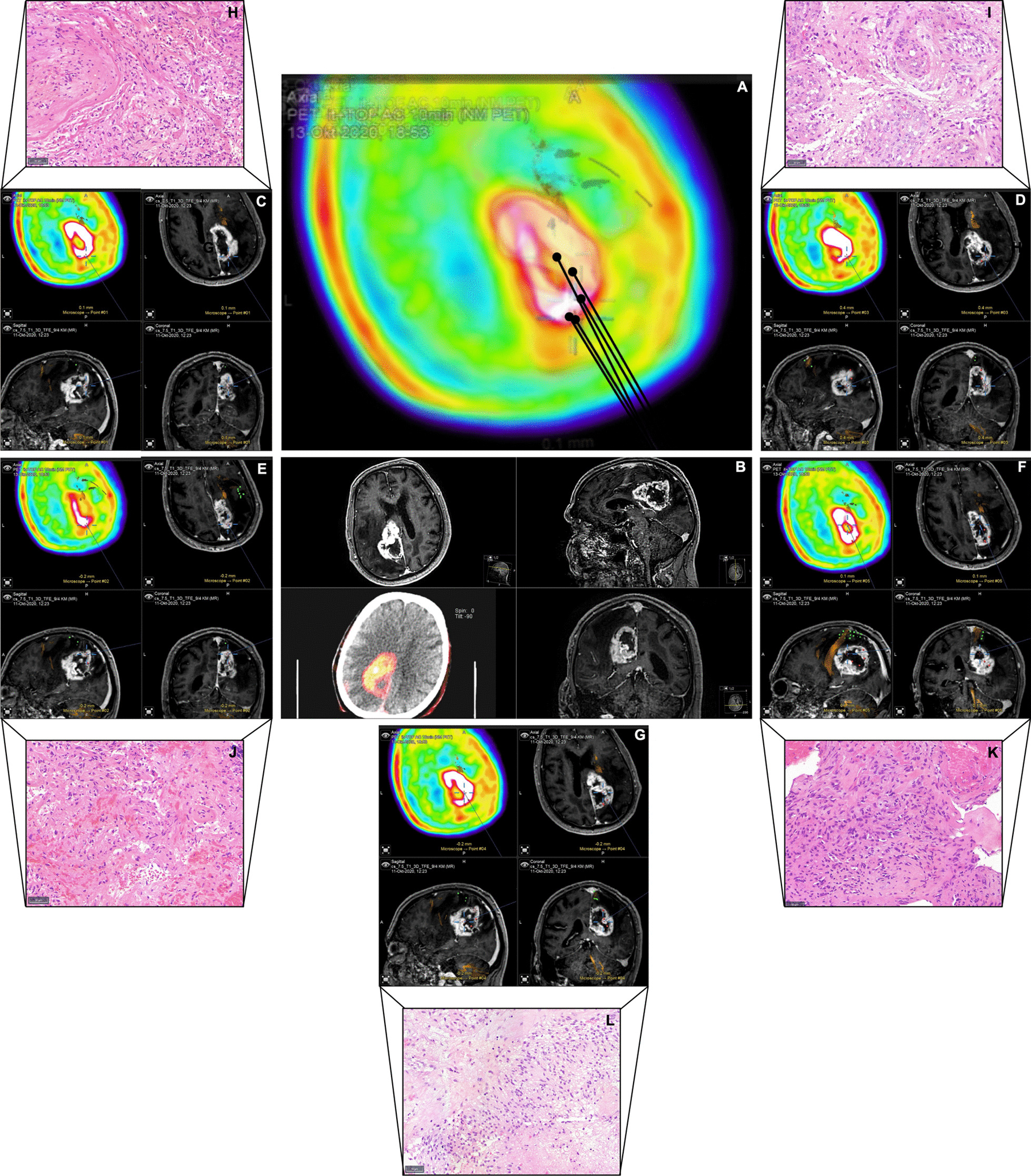
Visualizing of biopsy location and corresponding histology—an example. In **a**, a fusion of the FET-PET images with biopsy areas is depicted for better visualization. **b** Shows the preoperative MRI in all planes as well as the PET-CT. In **c**–**g**, all biopsy locations for one patient are marked with an extract of corresponding histology (**h**–**l**, scale bars 20 μm). **g** and **h** show high neovascularization, **i**–**k** low vascularization

Results of correlation analysis and ANOVA are summarized in Table 1. Figure 1 visualizes associations between imaging parameters and neovascularization. In Fig. 2, an example of the biopsy locations and the corresponding histology of one patient is depicted.

**Table 1:** Results of correlation

	Neovascularization		Cellularity	
	ANOVA F	*p*	Spearman’s *ρ*	*p*
[18F] FET PET	0.294	0.746	**0.229**	**0.039**
CBV	**6.055**	**0.003**	0.129	0.106
FA	1.55	0.215	− 0.095	0.231
MD	0.095	0.909	− 0.154	0.050

Bold values indicate significance

## Discussion

Combining a variety of imaging modalities has become an essential tool for assessment of tumor biology in GBM. In our study, biopsy target areas were selected based on [18F] FET PET, CBV, FA and MD. The 162 targeted biopsies of 43 patients with newly diagnosed, therapy-naïve GBM were then histopathologically analyzed for cellularity and neovascularization. Through this spatially precise targeting of tumor subvolumes, correlation between imaging and histopathology is robust, especially by taking into account the presence of intratumoral heterogeneity.

In our study, we observed a significant correlation between [18F] FET PET and cellularity, which is in line with previous studies [14, 15]. The increased demand for amino acids in proliferating cells might explain this association. Additionally, tracer uptake is believed to result from specific LAT amino acid transportation, but also from non-specific uptake due to various processes leading to increased permeability of the blood–brain-barrier, including pathological vascular proliferates [16]. Our results show a merely weak association between [18F] FET PET intensity and neovascularization. As opposed to some previous studies reporting a stronger correlation between tracer uptake and neovascularization [2, 15], we have highly focused on contrast-enhancing tumor, which might explain this discrepancy.

Our study detected a significantly increased CBV in areas with high levels of neovascularization, which is in line with previous studies [17]. Additionally, CBV showed a weak trend towards positive correlation with cellularity. This triangle association is feasible as rapid cell proliferation and resulting high cell density leads to hypoxic condition which triggers angiogenesis [18].

Furthermore, we detected a borderline negative correlation of MD and cellularity, which is contrary to the results of Stadlbauer et al. [19] who showed an positive correlation by including 77 stereotactic biopsies originating from 20 patients. On the other hand, Sadeghi et al. could not confirm this inverse correlation [17].

Main limitation of this study are missing imaging modalities of a subset of patients, especially missing [18F] FET PET data, thus explaining the lower power for finding significant associations between [18F] FET PET and histopathological characteristics. Non-simultaneous acquisition of [18F] FET and MRI is a further limitation. [18F] FET PET imaging included PET CT and PET MRI. On the other hand, this makes our results more generalizable. Another limitation is that several samples were not independent, since they were retrieved from the same individuals. Regarding correlating imaging parameters with histopathological analysis, we consider this aspect as neglectable, though.

Lastly, neovascularization was scored semiquantitatively by a single neuropathologist. Even if this might impede independent reproducibility, it avoids interobserver variance in our study, as described previously [2, 13].

## Conclusion

Our study confirms the important association of multimodal imaging with key histopathological characteristics of glioblastoma and highlights the potential of imaging to resolve spatial heterogeneity in these tumors. Our study also showcases the importance of interdisciplinary collaboration to unravel the association of imaging and tumor biology.

## Data Availability

All data is available on reasonable request addressed to the corresponding author.

## Abbreviations

ANOVA: Analysis of variance
CBV: Cerebral blood volume
CSF: Cerebrospinal fluid
CT: Computed tomography
DTI: Diffusion tensor imaging
DSC: Dynamic susceptibility contrast
FA: Fractional anisotropy
GBM: Glioblastoma
GM: Gray matter
IDH: Isocitrate dehydrogenase
MD: Mean diffusivity
MRI: Magnetic resonance imaging
[18F] FET: O-(2-[18F]fluoroethyl)-L-tyrosine
PET: Positron emission tomography
PWI: Perfusion-weighed imaging
rCBV: relative CBV
WM: White matter
WHO: World Health Organization
